# Supplementary figures and images for: A single dose of inactivated influenza virus vaccine expressing COBRA hemagglutinin elicits broadly-reactive and long-lasting protection
Source: PLoS One. 2025 Feb 21;20(2):e0308680. doi: 10.1371/journal.pone.0308680 (PMC11844911; doi:10.1371/journal.pone.0308680)

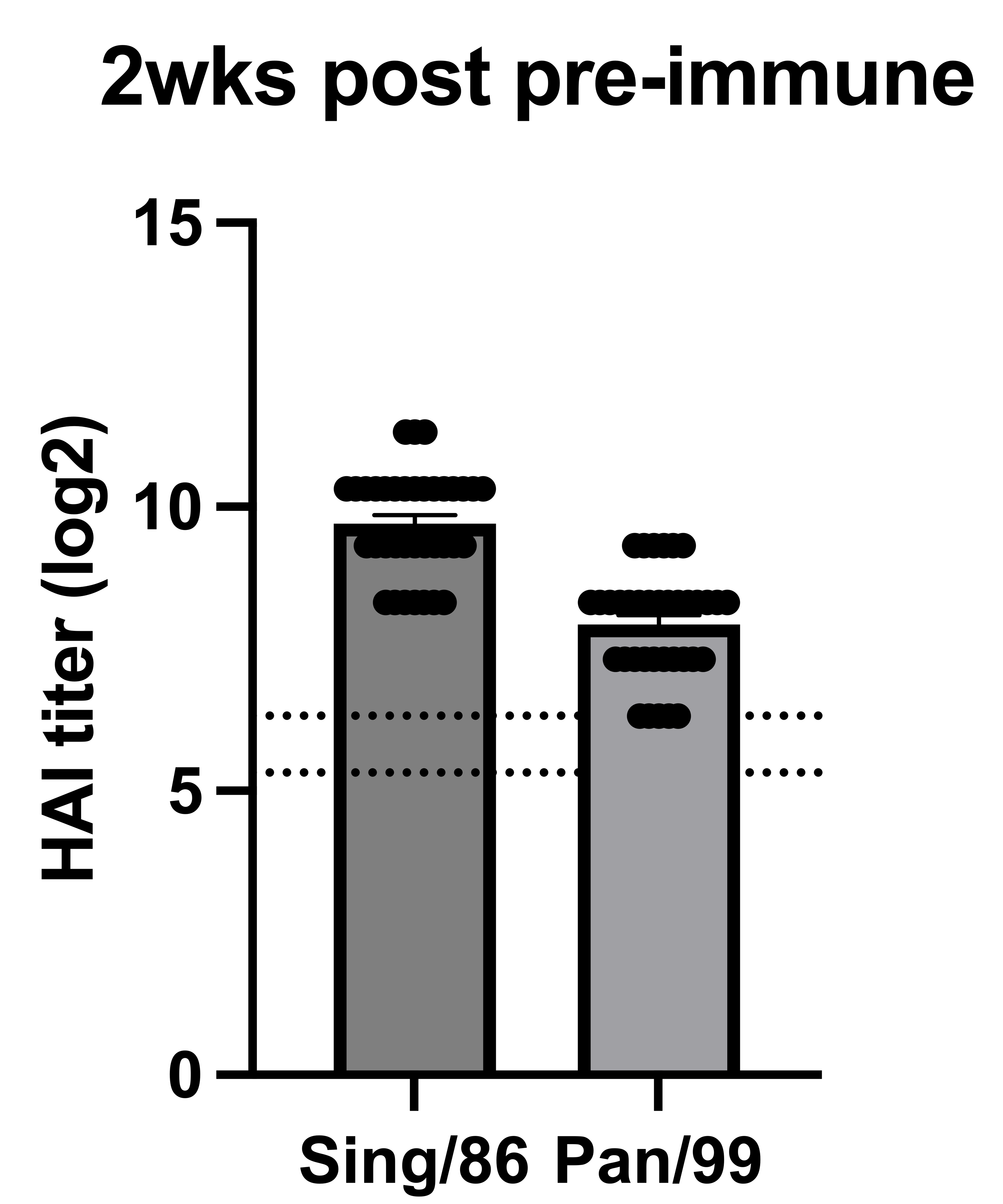

Supplement: S1 Fig — Sera samples collected 2 weeks post-pre-infection were collected and tested against the historical strains (Sing/86 and Pan/99). The X-axis represents the different strains. The Y-axis represents the Log2 HAI titers with absolute mean values ± SEM. The lower dotted line indicates the HAI titer of 1:40, and the upper dotted line indicates 1:80. (TIFF) [file pone.0308680.s001.tiff]

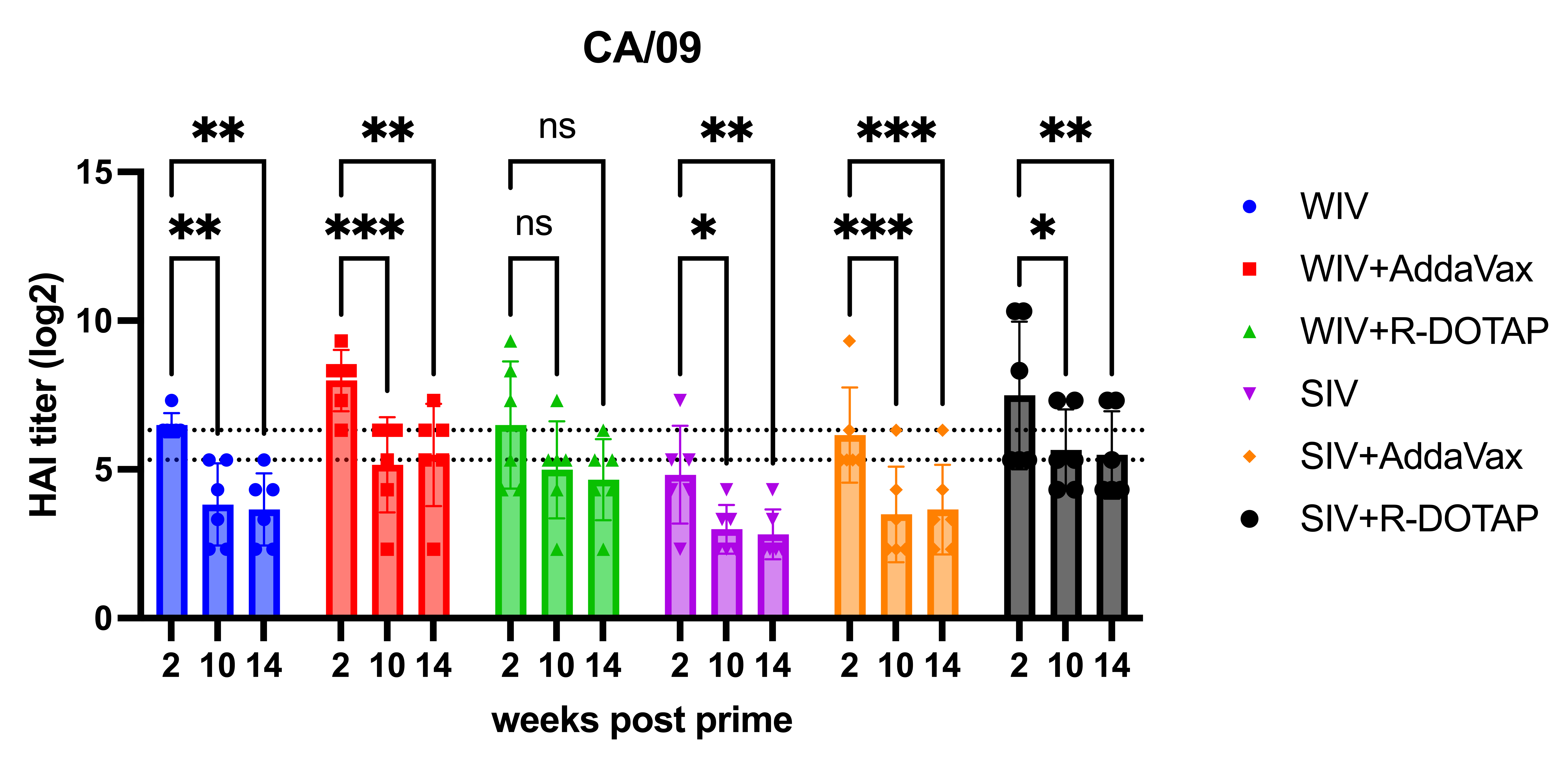

Supplement: S2 Fig — Sera samples collected 2, 10, and 14 weeks post-vaccination were tested against the CA/09. The X-axis represents the different sampling time points. The Y-axis represents the Log2 HAI titers with absolute mean values ± SEM. The legend shows the different vaccine groups. The lower dotted line indicates the HAI titer of 1:40, and the upper dotted line indicates 1:80. A P value of less than 0.05 was defined as statistically significant (*, P < 0.05; **, P < 0.01; ***, P < 0.001; ****, P < 0.0001). (TIFF) [file pone.0308680.s002.tiff]

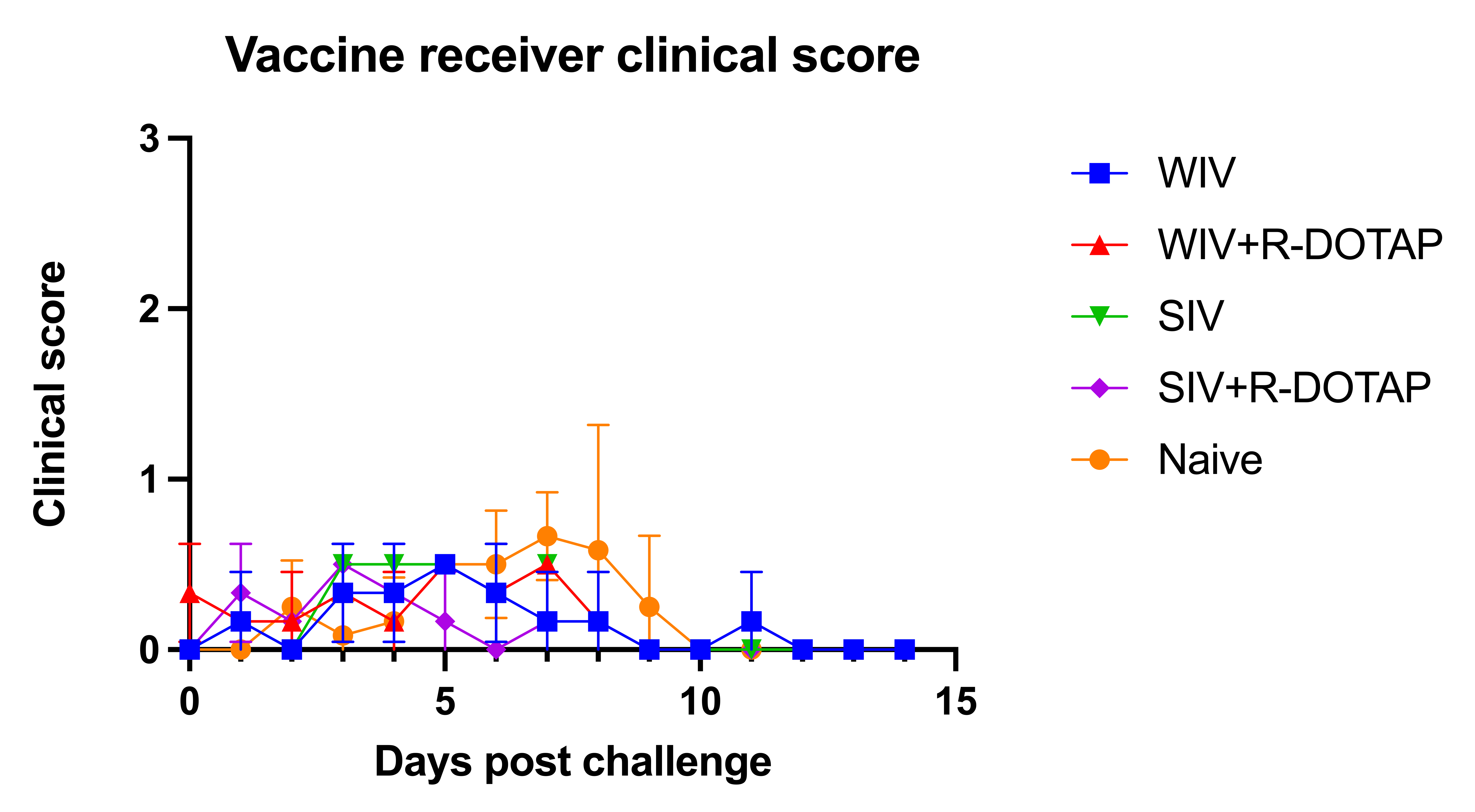

Supplement: S3 Fig — The legend shows the vaccine groups. (TIFF) [file pone.0308680.s003.tiff]

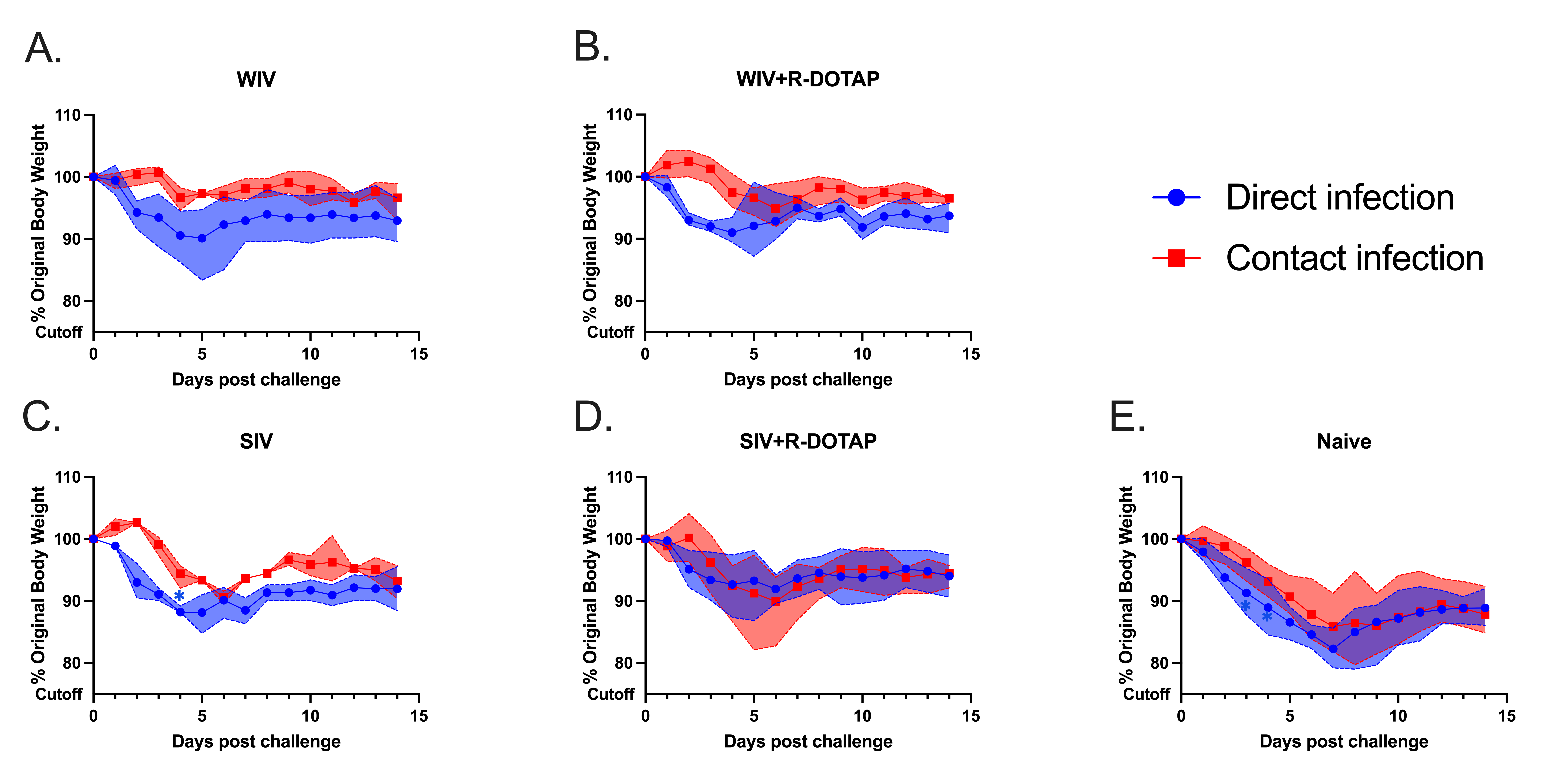

Supplement: S4 Fig — A: WIV-vaccinated ferrets. B. WIV+R-DOTAP-vaccinated ferrets. C: SIV-vaccinated ferrets. D. SIV+R-DOTAP-vaccinated ferrets. E. Naïve control ferrets. The blue area represents the body weight loss range post-direct infection. The red area represents the body weight loss range post-contact infection. (TIFF) [file pone.0308680.s004.tiff]
