# Supplementary material for: A single dose of inactivated influenza virus vaccine expressing COBRA hemagglutinin elicits broadly-reactive and long-lasting protection
Source: PLoS One. 2025 Feb 21;20(2):e0308680. doi: 10.1371/journal.pone.0308680 (PMC11844911; doi:10.1371/journal.pone.0308680)
Supplement: S1 Table — (DOCX) [file pone.0308680.s005.docx]

**Supplemental Table 1. Individual animal antibody levels, body weight loss, clinical scores, survival days, and viral titers in nasal washes post-infection.** HAI, hemagglutination inhibition; MN, microneutralization assay.

| Ferret ID | Vaccine | 14 wks post-vax CA/09-specifc | | Infection type | Lowest body weight | Max clinical score | Survival days | Viral shredding (PFU/ml) Log10 | | | | |
| --- | --- | --- | --- | --- | --- | --- | --- | --- | --- | --- | --- | --- |
|  |  | HAI titer (Log2) | MNA titer |  |  |  |  | 1DPI | 2DPI | 3DPI | 4DPI | 5DPI |
| T1995 | WIV | 5.32 | 80 | Direct infection | 94.48% | 0.5 | >14DPI | 5.04 |  | 3.72 |  | Undetectable |
| T1996 | WIV | 6.32 | 20 | Direct infection | 83.37% | 0.5 | >14DPI | 6.82 |  | 4.72 |  | Undetectable |
| T1980 | WIV | 6.32 | 80 | Direct infection | 92.33% | 0.5 | >14DPI | 5.92 |  | 4.20 |  | Undetectable |
| T1978 | WIV+AddaVax | 2.32 | 20 | Direct infection | 91.71% | 0.5 | >14DPI | 6.54 |  | 4.45 |  | Undetectable |
| T1979 | WIV+AddaVax | 5.32 | 80 | Direct infection | 85.41% | 0.5 | >14DPI | 6.78 |  | 4.53 |  | Undetectable |
| T1284 | WIV+AddaVax | 6.32 | 80 | Direct infection | 97.73% | 0.5 | >14DPI | 5.96 |  | 3.93 |  | Undetectable |
| T372 | WIV+AddaVax | 6.32 | 320 | Direct infection | 91.71% | 0.5 | >14DPI | 5.89 |  | 4.22 |  | Undetectable |
| T1973 | WIV+AddaVax | 7.32 | 160 | Direct infection | 95.85% | 0.5 | >14DPI | 6.33 |  | 3.04 |  | Undetectable |
| T1975 | WIV+AddaVax | 5.32 | 40 | Direct infection | 93.09% | 0.5 | >14DPI | 5.78 |  | 2.90 |  | Undetectable |
| T1281 | WIV+R-DOTAP | 4.32 | 80 | Direct infection | 89.90% | 0.5 | >14DPI | 6.30 |  | 3.22 |  | Undetectable |
| T127/ T370 | WIV+R-DOTAP | 4.32 | 160 | Direct infection | 87.19% | 0.5 | >14DPI | 5.90 |  | 3.29 |  | Undetectable |
| T376 | WIV+R-DOTAP | 5.32 | 320 | Direct infection | 92.86% | 0.5 | >14DPI | 6.24 |  | 3.28 |  | Undetectable |
| T1989 | SIV | 2.32 | 20 | Direct infection | 84.79% | 1 | >14DPI | 6.69 |  | 4.77 |  | 2.65 |
| T1990 | SIV | 2.32 | 10 | Direct infection | 88.65% | 0.5 | >14DPI | 6.10 |  | 3.66 |  | 2.40 |
| T1976 | SIV | 4.32 | 40 | Direct infection | 86.34% | 0.5 | 7DPI | 5.49 |  | 3.98 |  | 1.48 |
| T1984/403 | SIV+AddaVax | 2.32 | 80 | Direct infection | 92.14% | 0.5 | >14DPI | 7.45 |  | 5.03 |  | 2.22 |
| T1985 | SIV+AddaVax | 4.32 | 80 | Direct infection | 89.73% | 0.5 | >14DPI | 6.30 |  | 3.41 |  | Undetectable |
| T373 | SIV+AddaVax | 3.32 | 80 | Direct infection | 85.20% | 0.5 | >14DPI | 7.16 |  | 4.87 |  | Undetectable |
| T395 | SIV+AddaVax | 2.32 | 80 | Direct infection | 81.36% | 0.5 | >14DPI | 6.48 |  | 4.72 |  | 1.48 |
| T1974 | SIV+AddaVax | 6.32 | 640 | Direct infection | 85.05% | 0.5 | >14DPI | 6.91 |  | 4.20 |  | Undetectable |
| T1988 | SIV+AddaVax | 3.32 | 80 | Direct infection | 94.89% | 0.5 | >14DPI | 6.87 |  | 4.04 |  | Undetectable |
| T377 | SIV+R-DOTAP | 7.32 | 80 | Direct infection | 98.12% | 0.5 | >14DPI | 5.84 |  | 3.27 |  | Undetectable |
| T1983/402 | SIV+R-DOTAP | 5.32 | 20 | Direct infection | 94.26% | 0.5 | >14DPI | 5.71 |  | 3.20 |  | Undetectable |
| T1997 | SIV+R-DOTAP | 4.32 | 40 | Direct infection | 86.84% | 1 | >14DPI | 6.71 |  | 4.10 |  | Undetectable |
| T1982 | WIV | 4.32 | 20 | Contact infection | 93.01% | 0.5 | >14DPI |  | 3.58 |  | 5.63 |  |
| T1986 | WIV | 2.32 | 160 | Contact infection | 95.24% | 0.5 | >14DPI |  | 2.85 |  | 5.07 |  |
| T1987 | WIV | 4.32 | 40 | Contact infection | 97.07% | 0.5 | >14DPI |  | Undetectable |  | 4.98 |  |
| T1981 | WIV+R-DOTAP | 6.32 | 320 | Contact infection | 96.80% | 0.5 | >14DPI |  | 4.82 |  | 4.43 |  |
| T1993 | WIV+R-DOTAP | 5.32 | 160 | Contact infection | 91.97% | 0.5 | >14DPI |  | 4.73 |  | 4.95 |  |
| T1994 | WIV+R-DOTAP | 2.32 | 160 | Contact infection | 93.84% | 0.5 | >14DPI |  | 2.70 |  | 5.41 |  |
| T1977 | SIV | 3.32 | 20 | Contact infection | 91.58% | 0.5 | >14DPI |  | 3.04 |  | 6.20 |  |
| T1991 | SIV | 2.32 | 10 | Contact infection | 90.48% | 0.5 | >14DPI |  | 2.40 |  | 5.19 |  |
| T1992 | SIV | 2.32 | 20 | Contact infection | 89.52% | 0.5 | >14DPI |  | 5.21 |  | 5.57 |  |
| T863 | SIV+R-DOTAP | 7.32 | 320 | Contact infection | 93.82% | 0.5 | >14DPI |  | 2.97 |  | 5.08 |  |
| T1269 | SIV+R-DOTAP | 4.32 | 10 | Contact infection | 82.12% | 0.5 | >14DPI |  | 5.29 |  | 4.06 |  |
| T1280 | SIV+R-DOTAP | 4.32 | 40 | Contact infection | 93.22% | 0.5 | >14DPI |  | 5.89 |  | 5.26 |  |
| T1469 | N/A | 2.32 | 5 | Direct infection | 83.33% | 0.5 | >14DPI | 5.96 |  | 5.20 |  | 4.12 |
| T300 | N/A | 2.32 | 5 | Direct infection | 83.73% | 0.5 | >14DPI | 5.30 |  | 5.28 |  | 3.77 |
| T1533 | N/A | 2.32 | 5 | Direct infection | 79.22% | 2 | 7DPI | 5.73 |  | 4.95 |  | 5.11 |
| T3958 | N/A | 2.32 | 5 | Direct infection | 82.42% | 0.5 | >14DPI | 6.60 |  | 5.06 |  | 4.23 |
| T3966 | N/A | 2.32 | 5 | Direct infection | 79.48% | 2 | 7DPI | 5.85 |  | 5.03 |  | 4.48 |
| T3967 | N/A | 2.32 | 5 | Direct infection | 83.59% | 0.5 | >14DPI | 6.26 |  | 5.24 |  | 5.01 |
| T1468 | N/A | 2.32 | 5 | Contact infection | 85.41% | 1 | >14DPI |  | 6.26 |  | 5.88 |  |
| T1470 | N/A | 2.32 | 5 | Contact infection | 79.70% | 2 | >14DPI |  | 5.48 |  | 5.01 |  |
| T1542 | N/A | 2.32 | 5 | Contact infection | 81.31% | 1 | >14DPI |  | 6.10 |  | 5.48 |  |
| T3961 | N/A | 2.32 | 5 | Contact infection | 87.17% | 0.5 | >14DPI |  | 4.04 |  | 5.83 |  |
| T3971 | N/A | 2.32 | 5 | Contact infection | 91.23% | 0.5 | >14DPI |  | Undetectable |  | 6.82 |  |
| T3969 | N/A | 2.32 | 5 | Contact infection | 84.26% | 1 | >14DPI |  | 5.98 |  | 5.88 |  |
